# New Tools for Syphilis Research

**DOI:** 10.1128/mBio.01417-18

**Published:** 2018-07-31

**Authors:** Sheila A. Lukehart

**Affiliations:** a Department of Medicine, University of Washington, Seattle, Washington, USA; b Department of Global Health, University of Washington, Seattle, Washington, USA

**Keywords:** infectious disease, sexually transmitted diseases, syphilis

## Abstract

Syphilis research has been severely limited by the necessity to propagate Treponema pallidum *in vivo* in rabbits. After decades of erroneous or irreproducible reports of cultivation of T. pallidum, the recent very convincing report of its successful long-term *in vitro* propagation opens numerous opportunities for development of genetic tools for studying pathogenesis and protein function, antigenic variation, and surface exposure of antigens. The possibility of more rapid isolation of new strains will expand our knowledge of this organism beyond the century-old Nichols strain.

## Abstract (precis)

Syphilis research has been severely limited by the necessity to propagate Treponema pallidum *in vivo* in rabbits. After decades of erroneous or irreproducible reports of cultivation of T. pallidum, the recent very convincing report of its successful long-term *in vitro* propagation opens numerous opportunities for development of genetic tools for studying pathogenesis and protein function, antigenic variation, and surface exposure of antigens.

## COMMENTARY

Syphilis is a challenging disease. For public health officials, there is considerable frustration because, despite a focused National Plan to Eliminate Syphilis from the United States, the United States, Canada, and Western Europe are currently witnessing unharnessed increases in syphilis cases. The number of reported cases of infectious primary and secondary syphilis has increased 2.5-fold in the past decade (1), defying control by traditional strategies of testing, treatment, and contact tracing. For clinicians, the myriad manifestations of Treponema pallidum infection and troublesome serological tests can make diagnosis problematic, and the inability of the standard therapy, benzathine penicillin G, to eliminate organisms sequestered in the central nervous system makes patient management controversial (2). For scientists studying T. pallidum in the laboratory, the most vexing problems have been the fragile nature of the organism and the need to propagate it by continuous passage in rabbits. Finally, there is good news.

The recent report from Edmondson, Hu, and Norris describes, for the first time, the long-term continuous propagation of T. pallidum *in vitro* (3). While this methodology is still cumbersome and time-consuming, it is a major step forward for investigators in the field of *Treponema* research. This accomplishment culminates many decades of work. Although erroneous or irreproducible reports of cultivation have peppered the literature since the identification of T. pallidum at the turn of the 20th century, substantive progress was first described in a series of papers by Fieldsteel, Cox, and Moecklii in the early 1980s (4–6). In this work, which was replicated soon thereafter by Norris (7), T. pallidum numbers increased by up to 50-fold, with survival of 1 to 2 weeks. Subculture was not possible, however, making this approach unacceptable as an alternative to propagation in rabbits. Even this limited success was based upon a careful foundation of studies by Fitzgerald, Fieldsteel, Sandok, Norris, and others in the mid-1970s (8–11) demonstrating the attachment of T. pallidum to eukaryotic cells *in vitro*, the prolongation of treponemal survival in this setting, and the beneficial effect of low-oxygen environments and reducing agents in the medium. These early findings informed the conditions described by Edmonson et al. in the current report.

I remember those days as a graduate student in the Miller lab at UCLA, where Tom Fitzgerald and Steve Norris would undertake long and tedious studies, involving many hours hunched over the dark-field microscope, counting actively motile, sluggish, and nonmotile treponemes repeatedly for many hours, sometimes through the night. In subsequent years, David Cox, after Howard Fieldsteel’s death, undertook independent studies (12–14) on T. pallidum cultivation (and he kindly shared his insight, expertise, and supportive fetal bovine serum with those who continued to poke away at this issue). As Edmondson et al. so generously acknowledge, this accomplishment builds on the solid foundation of earlier work.

T. pallidum has been one of the few known pathogenic bacteria that had evaded *in vitro* cultivation. It appears that now, with long-term survival of multiple strains, subculturing, and retention of pathogenicity clearly demonstrated, a quantum advance has been achieved. The ultimate goal continues to be axenic culture, which may prove formidable because of the reduced genome of T. pallidum. It is worth noting, however, that Mycoplasma genitalium, whose genome is 60% the size of T. pallidum’s, can be adapted to axenic growth (15, 16). Even before that goal is achieved for T. pallidum, the ability to propagate T. pallidum in cell culture will greatly enhance the studies that are possible with T. pallidum.

For decades, studies of bacterial pathogenesis have relied on the ability to mutate, delete, and control expression of individual bacterial genes. The development of these genetic tools for T. pallidum has been hampered by the inability to select for mutants *in vitro*, and those of us who have attempted antibiotic selection in rabbits know the frustration of failure after weeks or months of effort. One of the most exciting aspects of the ability to cultivate T. pallidum is its likely facilitation of the development of genetic tools for the syphilis organism. This would provide a means to interrogate the functions of the Tpr (T. pallidum repeat) family of proteins (17), the roles of other putative virulence factors in pathogenesis, and perhaps define the numerous hypothetical proteins contained in the T. pallidum genome (18).

Investigators who study proteins exposed on the surface of T. pallidum have been stymied by the ever-present rabbit antibody bound to the T. pallidum surface as the organisms are harvested from rabbit tissue, causing higher than desired background levels in surface labeling and opsonophagocytosis assays. Even immunosuppression of the rabbit during T. pallidum propagation does not completely eliminate this problem. Growth of T. pallidum in the absence of rabbit serum, as in the protocol reported by Edmondson et al., should eliminate this issue, possibly making these assays more sensitive for identification of surface-exposed antigens.

The discovery of the ability of TprK to undergo antigenic variation in seven defined variable (V) regions has potentially explained the mechanism by which later stages of syphilis develop, as well as the decades-long persistence of infection in the untreated host (19, 20). Careful studies by Giacani et al. have demonstrated that immune evasion is mediated by the antigenic variation of TprK *in vivo* (21). Some strains, however, are much more likely to undergo V region variation than other strains. Specifically, the Nichols strain, which has been passaged continually in rabbits for over a century and is used almost exclusively by the majority of syphilis labs, undergoes variation only under significant immunologic pressure *in vivo* (e.g., permitting the infection to last for up to 1 month, during which time the majority of treponemes have been cleared by the immune response) (22). In contrast, the Chicago strain, which has been passaged much less in rabbits, undergoes a relatively high level of V region variation during early infection in the rabbit (21). It is difficult to quantitate the rate of sequence variation *in vivo*, but this may now be possible *in vitro*. Growth *in vitro*, in the absence of an immune response, will permit the investigation of the inherent rate of V region variation in various strains of T. pallidum and may facilitate the identification of key selective epitopes and the mechanism of immune selection.

It is expected that refinements will be made to the published cultivation method and that the *in vitro* system can be scaled down to microwells, as well as up to large flasks or even vats. These refinements will provide important new tools for the syphilis research community. Currently, we “clone” T. pallidum by intravenous inoculation of rabbits followed by biopsy of individual lesions on their backs, with subsequent propagation of T. pallidum from each biopsy specimen *in vivo*—this is a very lengthy process, involving a large number of rabbits (19, 23). The availability of *in vitro* culture of T. pallidum by limiting dilution in microwells may simplify this process. Similarly, new strains of T. pallidum are currently isolated by injection of the patient’s sample into a rabbit, followed by months of observation for development of antibody or clinical signs and then repeated passages *in vivo* to increase the number of organisms (23). Inoculation of microwells might permit the determination of successful isolation much earlier (e.g., by amplification of RNA by reverse transcriptase PCR), without the need for months of observation and the use of many rabbits.

To reduce the continuing burden of syphilis throughout the world, the ultimate goal of many treponemal laboratories is the development of an effective syphilis vaccine. This *in vitro* cultivation system for T. pallidum has brought us a large step closer to being able to develop the tools and do the experiments that are needed to understand the pathogenesis and host-pathogen interactions necessary for rational vaccine development.
